# Urinary Metabolic Markers of Bladder Cancer: A Reflection of the Tumor or the Response of the Body?

**DOI:** 10.3390/metabo11110756

**Published:** 2021-10-31

**Authors:** Greta Petrella, Giorgia Ciufolini, Riccardo Vago, Daniel Oscar Cicero

**Affiliations:** 1 Department of Chemical Science and Technologies, University of Rome “Tor Vergata”, 00133 Rome, Italy; petrella@scienze.uniroma2.it (G.P.); ciufolini@scienze.uniroma2.it (G.C.); 2 Urological Research Institute, San Raffaele Scientific Institute, 20132 Milan, Italy; vago.riccardo@hsr.it; 3 Department of Urology, Università Vita-Salute San Raffaele, 20132 Milan, Italy

**Keywords:** bladder cancer, metabolomics, diagnostic, prognostic, systemic response

## Abstract

This work will review the metabolic information that various studies have obtained in recent years on bladder cancer, with particular attention to discovering biomarkers in urine for the diagnosis and prognosis of this disease. In principle, they would be capable of complementing cystoscopy, an invasive but nowadays irreplaceable technique or, in the best case, of replacing it. We will evaluate the degree of reproducibility that the different experiments have shown in the indication of biomarkers, and a synthesis will be attempted to obtain a consensus list that is more likely to become a guideline for clinical practice. In further analysis, we will inquire into the origin of these dysregulated metabolites in patients with bladder cancer. For this purpose, it will be helpful to compare the imbalances measured in urine with those known inside tumor cells or tissues. Although the urine analysis is sometimes considered a liquid biopsy because of its direct contact with the tumor in the bladder wall, it contains metabolites from all organs and tissues of the body, and the tumor is separated from urine by the most impermeable barrier found in mammals. The distinction between the specific and systemic responses can help understand the disease and its consequences in more depth.

## 1. Metabolomics, Another Perspective in Clinical Biochemistry

*“Both the body and its parts are in a continuous state of dissolution and nourishment, so they are inevitably undergoing permanent change.”*

Ibn al-Nafis (1213–1288)

The word metabolomics comes from the ancient Greek, *meta-bàllo* (*μεταβάλλω*), which means to turn, change, transform. It combines two words: *bàllo*, a verb meaning to launch, and *metà*, meaning beyond. However, it was Aristotle’s work that conferred the word *metabolé* (*μεταβολή*) its scientific value, use and relevance. In the first book of Physics, he affirms: “Let us place as a basic assumption of our investigation that things that exist by nature, either all or some of them, are in motion: this is attested by experience” [1]. He also fixes in the word and the concept of *metabolé* that every movement is a change from something into something else. Therefore, for the ancient Greeks, the word *metabolé* represents the act of recognizing that change comes out from something that was hidden before. Therefore, in the original experience of Aristotelian science, the sense of metabolomics consists of considering nature as a place of continuous transformation in which the processes of change are not annulled but preserved by this tireless work of acquisition and overcoming. In transformation, what takes form preserves the progress in new and unexpected manifestations.

More than 2500 years after Aristotle’s biology studies, metabolomics is a broadly recognized scientific discipline that seeks to identify and quantify the entire collection of intracellular and extracellular metabolites (molecules with MW < 1500 Da), reflecting the permanent changes of the complex network of biochemical reactions in living systems to adapt themselves to the environment and pathological events. This includes measurements within biofluids like the serum, plasma, urine, cerebrospinal fluid, exhales, tissue, and biological extracts [2]. As a whole, Omics sciences such as genomics (genes), transcriptomics (mRNA), proteomics (proteins), and metabolomics (metabolites) adopt a holistic view for the study of how molecules compose a cell tissue or organism. The integrated study of all these techniques is what is called “systems biology”.

The need to characterize complex biochemical mixtures makes mandatory the use of state-of-the-art analytical techniques. In this way, it is possible to analyze compounds in all physical states, including solids (tissues, soil, and biological waste), liquids (biofluids, effluents, and water), and gases (breath, fumes, and scents). In addition, it is possible to perform in vivo or in vitro studies. All these applications are possible because metabolomics uses a wide range of instruments. In recent years, however, three technologies have proven to be the most widely employed: Nuclear Magnetic Resonance (NMR), Gas Chromatography-Mass Spectrometry (GC-MS), and Liquid Chromatography-Mass Spectrometry (LC-MS). These techniques allow quantification of various organic compounds, including lipids, amino acids, sugars, biogenic amines, and organic acids. Several reviews describe how they work and how they can be used in metabolomics [3,4,5,6]. Often the emphasis is on the differences and the relative advantages, but several studies demonstrate that it is more beneficial to consider them complementary [3,4,5,6,7,8].

Despite the ever-growing number of applications of metabolomics, the leading and most exciting ones refer to the clinical and biochemical fields. It hypothesizes how and when perturbations of metabolic pathways occur in phenotypic alterations and can represent a powerful tool in diagnostic or prognostic areas.

The central assumption about the cause of many chronic or severe diseases is that their origins are genetic. This paradigm has begun to change when recent epidemiological studies have shown that many causes of death and disease have environmental origins [9,10], along with the importance of the microbiome [11] and the epigenome [12]. Metabolomics provides evidence in favor of this new perspective since it contributes significantly to understanding of the cellular metabolism [9,13], the microbiome, and its effect on human health and disease [14,15]. Thus, metabolomics has helped identify many unexpected chemical causes in complex diseases like atherosclerosis, cancer, and diabetes [9,16]. Furthermore, these studies have revealed that metabolites (both endogenous and exogenous) play a more critical role in disease development, cell signaling, and physiological control than previously expected. For this reason, metabolomics can intervene in the diagnosis, prognosis, and prevention of many diseases, as many examples from the literature show [17].

## 2. Metabolomics and Cancer

Cancer is considered the result of genetic alterations at the nuclear and cytoplasmic levels of oncogenes and tumor suppressor genes. This theory is considered almost a dogma, to the point that the National Cancer Institute states that “Cancer is a genetic disease—that is, it is caused by changes to genes that control the way our cells operate, especially how they grow and divide”. In this context, the metabolic changes observed in cells, tissues, and organisms result from these genetic signatures and the deregulation of cellular energetics [18].

A different view gave rise to an alternative explanation of cancer as a disease that begins with mitochondrial metabolic dysfunction, in some sense returning to Otto Warburg’s original observation [19]. However, recent data indicate that mitochondrial metabolism can be down- or upregulated, classifying tumors into two types: oxidative and non-oxidative [20]. From this perspective, metabolic alterations are the cause of the genetic alterations that influence the development of cancer. What causes metabolic alteration is a matter of discussion, but potential candidates are: increased inflammation and ROS formation. Although we do not yet have enough evidence to fully embrace the metabolic theory or understand the relative importance that both factors, genetic mutations, and metabolic alterations, play in the origin of the disease, metabolomics can make a significant contribution. From a genetic point of view, cancer is a disease of extreme complexity since a quick calculation based on the number of genes associated with cancer and the number of combinations of mutations that become tumorous yields a figure of more than one million different cancer genotypes. This complexity is reduced when analyzing the metabolic alterations, usually limited to three pathways: aerobic glycolysis, glutaminolysis, and one-carbon metabolism [21]. In the meaningful summary by D. Wishart: “[…] the bottom line is that while cancer as a genetic disease looks to be impossibly complex, cancer as a metabolic disease appears to be remarkably simple” [21]. This scenario assigns metabolomics a unique advantage for discovering new specific therapeutic targets and diagnostic markers.

## 3. The Critical Challenge of Bladder Cancer Diagnosis

Bladder cancer (BC) is the most common urinary tract cancer and a leading cause of mortality worldwide, with approximately 550,000 new cases and 160,000 deaths per year [22]. The incidence of bladder cancer differs according to the geographical region considered: the age-standardized incidence (ASI) is one-third less in undeveloped with respect to high-developed countries. The World Health Organization (WHO) predicts a rise in cases and deaths for the near future due to increased life expectancy [23].

BC encompasses a wide range of histologies: urothelial carcinoma (UC), which represent the majority (~90–95%) of bladder tumors, squamous cell carcinoma (SCC) (2–5%), adenocarcinoma (0.5–2%), and small cell carcinoma (<1%). BC’s risk factors include occupational factors, age, sex, race, socioeconomic status, personal health, diet, and infection by pathogens [24,25,26]. BC tumors are divided into two classes depending on whether they invade the detrusor muscle (muscle-invasive bladder cancer, MIBC) or not (non-muscle-invasive bladder cancer, NMIBC). The first presents a higher risk of metastasis of lymph nodes or other organs but, fortunately, represents only 25% of diagnosticated BC cases [23]. NMIBC generally involves the fibroblast growth factor receptor 3 (*FGFR3*) mutation, producing cancer with a high recurrence rate but a low risk of progression. By contrast, MIBC and carcinoma in situ exhibit deletions or mutations of *TP53*, RB transcriptional corepressor 1 (*RB1*), erb-b2 receptor tyrosine kinase 2 or *PTEN*, leading to metastatic cancer [27]. A link between some of these genotypes and cell phenotypes was recently observed, leading to the result that cell lines associated with a low risk of progression present an activated oxidative metabolic state, while those associated with a high risk present a non-oxidative state and high glycolytic activity [28].

Evaluation of patients suspected of having BC is performed using cystoscopy, an invasive endoscopic procedure performed with a flexible scope and with local anesthesia [29]. Histological evaluation is required if reddish flat papillary or solid lesions are observed because benign conditions like inflammatory diseases can mimic BC. Trans Urethral Resection of Bladder Tumor (TURBT) or resection of the entire area is used to obtain information about the histology of tumors. In addition, an inspection of cells in the urine (cytology) can be performed to detect missed cancer. Cells with a malignant appearance indicate cancerous lesions in the bladder and warrant cystoscopy and histological investigation.

## 4. Metabolomics of Bladder Cancer: Three Open Questions

The introduction of metabolic markers for an accurate diagnosis of BC and its risk of progression may decrease disease management costs and increase patients’ quality of life [30]. The availability of non-invasive markers for diagnosis would also improve patients’ susceptibility to routine screening, thereby increasing the effectiveness of preventive diagnostics. Therefore, it is essential to render prevention non-invasive and thus more efficient, even without apparent symptoms. In addition, the biochemical interpretation of the metabolic unbalances that can result from these screenings can open new opportunities for development of more effective therapies and monitoring of treatment and disease evolution.

Metabolomics may be the most appropriate way to achieve this goal. In the particular case of BC, the direct contact of the tumor with urine makes it feasible that specific biomarkers can be present in this fluid. Many specific reviews have been published about urinary markers of BC, testifying to the great interest in this field [31,32,33,34,35,36,37,38,39,40,41]. We have revised most available data on urinary metabolomics and bladder cancer to answer three key questions: (i) is it possible to use the urinary metabolic profile to detect BC? (ii) In the case of a positive answer, what are the metabolites responsible for this difference? (iii) What is the origin of the observed metabolic imbalances?

### 4.1. Can We Diagnose Bladder Cancer by Analyzing the Urinary Profile?

Twenty-five papers about discovering BC diagnostical metabolic markers in urine have been published in the literature (Table 1). Almost all the studies have used MS coupled with gas or liquid chromatography for the quantification of metabolites. The relatively low number of works using NMR reflects the complexity of urine as a biofluid because it contains many compounds and highly variable composition.

In all cases, the authors have concluded that there is a significant difference between the urine metabolic profiles corresponding to a control group with respect to BC. This unanimous result indicates that urine is sufficiently sensitive to the metabolic changes caused by a tumor in the bladder. However, to be considered a biomarker of disease, its alteration needs to be specifically related to that illness. From this point of view, it is relevant that different studies have used control groups made up of individuals with other pathologies instead of healthy subjects.

An example is the attempt to distinguish the metabolic signature caused in urine by BC and kidney cancer (KC), the top-two-incidence urological cancers [49]. The authors were able to classify the control, BC, and KC groups with 100% specificity and sensitivity using multivariate analysis. In a successive study, the urinary profile was used to differentiate the metabolic profile of 138 patients with BC from that of a control group of 121 persons that included 52 patients with non-malignant hematuria (HU) [52]. This distinction is particularly pertinent since hematuria is a widespread condition in BC and could constitute a confounding factor in diagnosing this disease [23]. A more recent study demonstrated that it is possible to distinguish between BC and renal cell carcinoma (RCC) patients, both in the presence or absence of hematuria [64]. Peng et al. [53] found that ten metabolites were responsible for the significant distinction between BC patients and a control group formed by subjects with urinary tract infection (UTI) and HU. On the contrary, no urinary metabolite was found to differentiate BC from prostate cancer (PCa) [68].

### 4.2. Which Are the Metabolites Responsible for the Difference in the Urinary Profile?

If the first question has a unanimous affirmative answer, the markers proposed in each study are very different, most of the time, from each other, which prevents a clear consensus on which metabolites are responsible for the metabolic differentiation of urine from BC patients. The combined results of all the studies in Table 1 generate a list of 352 putative urine markers for the presence of BC, but only 20 (6%) were found in at least three studies to have a significantly altered level (Table 2). Even the metabolite with the most significant consensus, hippuric acid, showed a concentration change in less than half of the studies. The variation direction was also poorly reproduced: only eight metabolites (2%) of the list in Table 2 showed the same variation between control and BC groups among the different studies, further reducing the consensus list. If we consider only those markers proposed in at least three studies, the occurrence of BC will potentially cause a decrease in the levels of hippuric and citric acids and an increase in lactic acid, taurine, valine, glutamine, histidine, and erythritol.

These disappointing numbers are not unique characteristics of metabolomics applied to BC. For example, in PCa, thirteen studies published from 2015 to 2020 proposed a total of 179 different putative urinary biomarkers. Of these, only four (2%) were repeated in at least three studies showing the same variation [69]. This lack of consistency among the results is undoubtedly multicausal, and in the following chapters, we will address some of the potential problems affecting the reproducibility of results.

#### 4.2.1. Sample Size

One possible reason for the lack of reproducibility is the use of small cohorts in the experiments. The most frequently proposed limit of published metabolomics studies is the insufficient number of samples analyzed and, as a result, the number of population studies is still insignificant compared to the overabundance of pilot experiments. The appropriate sample size calculation is not easy in metabolic phenotyping studies because of their top-down hypothesis-free characteristic (the so-called untargeted approach), complicating the experimental setup [70]. Nevertheless, the lack of reproducibility in the results should be a potent incentive to improve the significance of the experimental results by recruiting more participants.

#### 4.2.2. Geographical Origin, Economic Status, and Diet

The heterogeneity among the different studies regarding the participants’ geographic origins, economic status, and diet may contribute to the distinct metabolic alterations observed. Urine’s metabolic composition is strongly dependent on lifestyle and diet [71,72,73], two factors highly related to the country and even the different cities where samples are collected [72]. The analysis of 2732 urine samples from 1391 subjects across five European countries revealed systematic variation in the metabolic profiles, especially in terms of gender, country, and, to a lesser extent, economic status [71]. Even if socioeconomic status’s effect was generally less marked, the two primary metabolite variations associated with this factor are those of hippurate and citrate [71], two compounds among the most repeated as biomarkers of BC in the different studies (Table 2). The impoverishment in the quality and quantity of food consumed by populations at risk of poverty may to some extent influence these alterations [71], as was previously observed for Brazilian children [74]. The relationship with diet may become an important confounding factor that detracts from the observed variations concerning BC. For this reason, all the people participating in a metabolomics study must follow a standardized diet at least 24 h before collecting the samples [75].

#### 4.2.3. The Control Group

The definition of a proper control group is challenging, especially when used as a reference to BC patients since they constitute an elderly population with significant comorbidities. The higher incidence of BC in males is another characteristic to consider when defining the control group. Given that gender is one of the most critical determinants of urinary composition [76], results may be biased if this is not adequately considered. For example, the urinary citric acid concentration is more elevated in females [77], and this difference can partially explain the observed decrease of this metabolite level for the BC group when more males than the control group form it. The importance of matching both sex and age has already been pointed out in a metabolomics study of urinary BC biomarkers: tryptophan metabolism, the citrate cycle, and pantothenate and CoA biosynthesis heavily contribute to inter-individual variations. In this case, and to obtain meaningful results, the authors proceeded with cohorts strictly matched in age and sex [59].

#### 4.2.4. BC Heterogeneity

Disease heterogeneity is also a strong element that can lead to disparate results when looking at urinary biomarkers. BC presents one of the highest mutational burdens, only exceeded by lung and skin cancer [78]. This enormous genetic variability causes a vast heterogeneity that is manifested in at least five different levels: interpatient (different subjects present tumors with different genotypes); intratumoral (different spatial regions of the primary tumor do not share the same genetic alterations); intertumoral (differences in the genotype among multiple primary tumors or metastatic sites); circulation (difference between tissue-based and circulating markers) and temporal (genetic changes in the tumor over time and/or during treatment) [79].

In addition to the complexity and instability of the BC tumor genotype landscape, cancer cell metabolism itself is also highly variable. It is subject to environmental signals, mainly generated by the tumor microenvironment. Variations in the levels of oxygen and nutrients can induce metabolic heterogeneity, and the cell metabolic phenotype can further change during tumor progression because of a higher limitation in nutrients [80]. Different studies probably searched for biomarkers almost like they were characterizing distinct diseases due to all these changes in the genotype and phenotype. For example, in some studies, patients with NMIBC and MIBC were considered together, although their metabolic profiles are expected to be very different, as shown in tissue samples studies [81]. Three major pathways were found altered in MIBC, including increased eicosanoid signaling, enhanced de novo synthesis of NAD+, and increased heme catabolism. Even if a study considers only one of these two cancer categories, it is important to note that these broad classifications include carcinomas presenting different stages and thus representing a different phase of cancer progression. For example, it was observed that tryptophan metabolism is upregulated in the urine of high-grade NMIBC patients when compared with low-grade NMIBC patients [58]. In the study by Alberice et al., a series of markers for the BC diagnosis specific to the grade and stage were proposed [82]. The authors also considered cases of recurrence for patient classification and found prognostic markers specific to BC stability. The differences found in this work demonstrate that, as the control, the patient group chosen for a metabolomic study should also be homogeneous. Although it is still a pending issue in this field, a large cohort is the only way to mediate the heterogeneities mentioned above when searching for universal BC urinary biomarkers.

#### 4.2.5. Technical Issues about Biomarkers Identification and Quantification

The identification and quantification of metabolites in biofluids also face a series of technical difficulties. LC-MS is the most diffuse platform in these untargeted studies, and problems such as detector saturation and matrix effect can alter the signal intensity in particular samples [83]. Compound identification without the use of labeled standards is a further challenge. According to the Metabolomics Standard Initiative, this corresponds to level 2 identification (putative annotation); for MS, the scientific community agrees that a direct comparison of the experimental data with an authentic reference standard is essential for level 1 identification using MS data. Level 1 was only granted in very few cases among the studies in Table 1 [45,48,51], while in all other LC-MS-based analyses, metabolite structure determination was only putative and based on comparison with MS libraries or MS/MS data. This may add ambiguity about the actual chemical structure of the quantified features and can, in part, contribute to the lack of agreement among the different studies. A dataset composed of accurately identified and quantified metabolites yields more robust and, therefore, more comparable results across different studies for biochemical information and diagnostic/prognostic purposes. A possible way to reach this has been recently proposed by the synergic use of NMR and UHPLC-HRMS [83].

### 4.3. What Is the Physiological Origin of These Imbalances?

Although poorly reproducible, studies attempting to diagnose the presence of BC through urine have found significant alterations in the metabolic profile in this biofluid caused by the disease. However, the origins of these variations are still in debate. What is the relative weight of the systemic response vs. metabolite exchange facilitated by direct contact of the tumor with the urine? Determining which mechanism is the major contributor to the observed alteration is not crucial if the purpose is diagnostic, but it is essential to understanding the biochemical reason.

#### 4.3.1. The Direct Exchange between Tumor and Urine

Most of the studies cited in Table 1 embrace the “direct contact” theory, considering urine as a sort of liquid biopsy [43,44,46,49,52,53,54,55,58,59,60,61,62,63,66,67]. Therefore, all biochemical interpretations of the urinary metabolic variations are explained by unbalances in tumor cell pathways. This theory predicts an elevated exchange of metabolites across the uroepithelial membrane. Historically, the passage of substances from the urine into the inner layers of the bladder was the first conjecture used to explain the origin of the disease. In the late 19th century, the German physician Rehn noticed that the aniline dye industry workers showed an increased risk of developing this tumor [84], transforming BC into the first known chemically induced cancer. Urine concentrates carcinogens that can be inhaled, consumed, or absorbed through the skin, and they subsequently come into contact with the lining of the urinary tract [85]. All these substances have in common that they are or can be metabolized to highly reactive electrophilic compounds [86]. They subsequently attack electron-deficient sites in proteins and nucleic acids, forming covalent adducts or inducing mutagenesis [87].

The best strategy to understand whether the changes in urine and those occurring inside tumor cells are linked is to compare tissue and urine metabolomics results. So far, only two works regarding BC have explored these correlations. Putluri et al. found 25 metabolites altered in tissue and urine [47], whereas Alberice et al., using solid-state and solution NMR, discovered amino acids, glutathione, and taurine metabolic pathways significantly altered in both matrices [82].

The coincidence in tissue and urine alterations is insufficient to postulate the specificity of biomarkers. For example, decreased citrate levels and increased levels of leucine, valine, and taurine were observed contemporarily in tissue and urine samples of patients with PCa and were therefore postulated as specific biomarkers of this cancer type [69]. However, the same variation pattern was observed in the urinary metabolic profile of BC, as reported in Table 2 for citrate, valine, and taurine. An increase in leucine was also observed in the urine of BC patients in two studies [47,51]. Specificity can potentially arise from a different quantitative alteration of these metabolites in the two disease profiles. In this case, it will be necessary to accurately measure the biomarkers’ levels, increasing the experimental difficulty of the evaluation.

The number of studies comparing urine and tissue metabolic profiles is insufficient to determine whether changes in this biofluid reflect tumor metabolic imbalances preponderantly and thus whether metabolite exchange is sufficiently efficient. For this to occur, the chemical substances must pass through the most impermeable membrane in our body: the uroepithelium.

#### 4.3.2. The Impermeable Barrier between the Tumor and Urine

The uroepithelium constitutes the interface between the urinary space and underlying tissues. It is formed by different layers, from the superficial and highly differentiated multinucleated umbrella cells, several layers of intermediate cells, and a layer of basal cells. The paracellular diffusion of substances is prevented by the tight junctions connecting the umbrella cells, converting the uroepithelium into an impenetrable barrier to most substances present in urine [88]. Glycosaminoglycan (GAG) forms an extra negatively charged layer, further contributing to the urothelial barrier function [89]. These features generate the most resistant human barrier that shields the bloodstream from dangerous bacteria and high-risk substances.

The urine composition is highly different from plasma: urine osmolality ranges from 50 to 1200 mosmol/kg, against 280–290 mosmol/kg of blood; its pH lies between 4.5 and 10, compared to 7.4 of blood; and it contains high concentrations of ammonia, urea, and toxins. Urine must be stored for prolonged periods, and thus the function of the barrier is to prevent the passage of highly permeable molecules into the bloodstream that can cause harmful changes in osmolality, pH, and ionic strength [90]. In vitro measurement of the urothelium permeability established that urea, ammonia, water, and proton values were very low [91,92], suggesting that the in vivo bladder is an excellent barrier preventing these substances’ movement from urine to blood [93].

Tumor formation can potentially change this scenario by structurally altering the luminal surface membrane. For example, the neoplastic cells may fail to differentiate during chemical carcinogenesis, producing a modified membrane with no hexagonal sub-structure [94] and a concurrent increase in ion permeability [95]. This modification can hypothetically be more important for luminal MIBC, as it was suggested that this cancer subtype might arise from the transformation of umbrella cells [96]. However, luminal tumors show significant basal–luminal plasticity, suggesting that all bladder cancers arise from basal stem cells [96].

#### 4.3.3. Is the Tumor–Urine Metabolic Exchange Enough?

For a series of reasons, it is difficult to convincingly explain most of the altered urinary metabolic profile exclusively invoking the “direct contact” theory. Studies carried out with drug candidates for intravesical treatment suggest that the permeability of the uroepithelium remains low for most substances, even in the presence of a bladder tumor. This type of treatment takes advantage of the direct access of therapeutic agents to the bladder provided by the urethra, maximizing the exposure of the tumor to them and at the same time limiting the toxicity of the drug by decreasing systemic exposure [97]. Shen et al. determined the ratio between the urine concentration and that in the interface between the urothelium and the submucosa for different drugs [97]. Compounds with marked lipophilicity showed values up to 0.5, but more polar compounds like mitomycin c, doxorubicin, and 5-fluorouridine showed ratios of 0.02–0.03. These results and others in the literature [98,99] indicate that lipophilicity is a key determinant of drug penetration through the urothelium. Most of the compounds listed in Table 2 that are small and hydrophilic do not possess the ideal characteristics needed to cross the uroepithelial membrane efficiently. To reinforce this concept, it should be noted that enhancers are frequently necessary to permeate the drugs used in intravesical treatment adequately. The most promising ones are liposomes, surfactants, nanogels, EMDA, RITE, and low-energy shock-wave therapy [100]. These strategies underline that a highly efficient exchange of compounds between the bladder cavity and the tumor is not warranted by BC’s mere presence.

Moreover, a significant influence of tumor cell metabolism on urine composition is more plausible in animal models in which the tumor-to-host mass ratio is often large than in humans, with a lower proportion between the masses of the tumor and the whole organism [101]. At the same time, the tumor influence is expected to be higher for those metabolites that show a small mass variation in the urine composition. This quantity can be calculated from the mean urine concentration and the fold change measured in the different studies, as shown in Table 3. A first analysis shows that the most altered metabolites in BC patients are also among the most concentrated in the urine. This result is surprising considering that almost all studies used MS, a technique that, thanks to its high sensitivity, does not limit the detection of markers to highly concentrated compounds. If we add the high fold changes proposed for their alteration in BC, we conclude that the variation in terms of mass must be significantly high to explain the predicted results. For example, to decrease the amounts of hippurate and citrate in the urine to the extent observed on average, tumor cells should consume 100 and 40 mg, respectively, between two urine voids. Similar large variations are expected for most of the metabolites of the consensus list (Table 3).

The presence of an efficient barrier separating tumor and urine and the low tumor-to-host mass ratio are two eloquent reasons to question the impact of the “direct contact” theory. Another mechanism is more likely to contribute to the substantial mass changes listed in Table 3. The most obvious one involves the systemic response. In fact, many of the first studies in humans could not find significant metabolic changes in patients with early-stage and localized cancer, reinforcing the possibility that the variations observed in most of the subsequent studies were not because of cancer per se but a manifestation of a phenotype determined by systemic responses [101]. The inclusion of the whole body to explain the changes produced in urine by BC goes in the direction recently suggested by Doru Paul, who invites to replace the “tumoricentric” paradigm with a new one that focuses on the whole “cancerized” organism [102].

## 5. The Systemic Response of the “Cancerized” Organism

A key concept that helped conceptualize the metabolic changes induced by cancer in the whole organism was proposed by Al-Zhoughbi et al., the so-called tumor macroenvironment [103,104]. According to the authors, the tumor environment constantly changes due to three types of interactions, differing by the type of cells sending and receiving the signal and the spatial distribution of the cellular signaling. The first type regards ligands released by cancer cells binding to receptors on their own surface. A second paracrine interaction involves local growth factors and inhibitors, defining the tumor microenvironment. The third type requires endocrine signaling through the neovasculature formed by angiogenesis and is a critical step in cancer growth and progression. This mechanism, involving the macroenvironment, allows cancer cells to interact with other organs and systems [103,104].

**Scheme 1:**
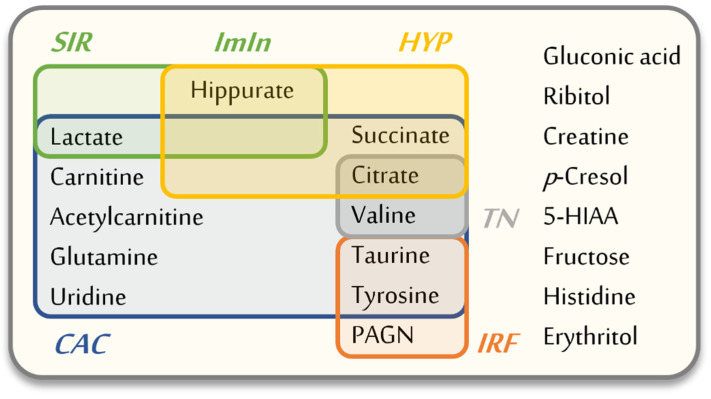
Proposed BC urinary biomarkers and their potential involvement in systemic responses. Abbreviations: CAC: cachexia, HYP: hypertension, ImIn: immunity inhibition, IRF: impaired renal function, SIR: systemic inflammation response, TN: thrombosis network, 5-HIAA: 5-hydroxyindoleacetic acid, PAGN: phenylacetylglutamine.

The endocrine signaling explains how the whole organism participates with the tumor to develop the disease. To this end, a complex systemic pathogenic network develops, allowing communication between separated cancer tissues and the rest of the organism. This network creates a co-dependence, and the interaction between the tumor and the whole organism induces the appearance of cancer-induced systemic pathologic networks (CISPN) [102]. A list of six systemic hallmarks, each established through a different CISPN, has been recently proposed which includes the connection between the primary tumor, the bone marrow and the distal metastasis, the global inflammation, the immunity inhibition, the metabolic changes leading to cachexia, the propensity to thrombosis, and the neuroendocrine changes [102]. The following chapters will review some of them and different comorbidities commonly associated with BC. In all cases, we have tried to summarize the results that different metabolomics studies have found, and we have analyzed which proposed markers for BC may be altered due to the different systemic responses (Scheme 1). This kind of crosscheck is particularly important since patients with BC represent a highly comorbid population, given their age and a generally high prevalence of other diseases, smoking, and poor performance status [105].

### 5.1. Systemic Inflammation: Hippurate and Lactate

A large body of evidence correlates systemic inflammatory response (SIR) with poor oncologic outcomes in cancer patients. SIR-related hematological biomarkers are considered a potential source of information about risk stratification for recurrence and mortality of patients with BC [106]. One of these biomarkers is the absolute neutrophils to absolute lymphocytes ratio (NLR), which significantly correlates with disease recurrence and progression in NMIBC [107], and even higher NLR values were observed in MIBC patients compared with NMIBC patients [108].

The coexistence between BC and a systemic inflammatory status undoubtedly has consequences at the level of the urinary metabolic profile. One of these is host dysbiosis [109], which alters the intestinal microbiota composition and ultimately affects urinary hippurate content [65,110]. This modification may be one of the main reasons for its decreased concentration observed in BC patients’ urine [44,46,47,48,49,56,60,61,62,63,65], as already observed for inflammatory bowel diseases (IBD), Chron’s disease, and ulcerative colitis [111]. Moreover, the hippurate excretion level has been associated with the functional state of other systems, including excretory, cardiovascular, and neurological systems [112]. Not surprisingly, urinary hippurate was found to be significantly altered in 20 types of cancer and can be safely considered the least specific metabolite by tumor type [113].

Lactate increase in BC urine [50,51,56,61,63] can also be connected with SIR. Pietzner and coworkers found a positive correlation between urinary lactate and white blood cell count (WBC) [114], one of the laboratory markers used in clinical practice to assess the degree of inflammation. The hypoxic conditions and the high energetic demand of proliferation of immune cells cause lactate to accumulate at sites of inflammation [76]. This metabolite also modulates the pro-inflammatory response of T cells and induces the trapping of these cells at the site of inflammation [115].

### 5.2. Immunity Inhibition: Hippurate and Lactate

The inhibition of the immune system and the induction of inflammation are two determining factors for tumor growth and progression [116]. Metastatic tumor cells release exosomes containing programmed death-ligand 1 (PD-L1) on their surface. Stimulation with IFN-γ increases PD-L1 levels that suppress CD8 T cell function and facilitate tumor growth [116]. In the specific case of BC, several trials have shown encouraging efficacy of checkpoint inhibitors, all targeting the PD1/PD-L1 pathway [117]. However, more than half of the patients with an advanced disease did not show clinical benefit with this type of therapy [118]. It is, therefore, necessary to find markers that can anticipate the response to immunotherapy.

As for inflammation, there is mutual interference between the immune system and the gut microbiome [119,120]. This relationship has led to the suggestion that the microbiota and/or its metabolites may influence the efficacy of immune checkpoint inhibitors [121,122,123,124]. The work by Hatae et al. proved that hippurate and other three metabolites derived from the microbiome are quite valuable when anticipating a high response in patients with non-small cell lung cancer [125]. Although there are no studies on this subject, it is possible that the urinary level of hippurate of BC patients could be used in the future to anticipate their response to immunotherapy.

Several groups have highlighted the role of lactate as an immunosuppressant in cancer patients. Increased lactate levels are the main factor in the tumor microenvironment acidosis. Decreased pH value in the extracellular environment impairs the functions (activation, cytotoxicity, chemotaxis, motility, and proliferation) of CD8+ and CD4+ lymphocytes [126,127]. The mechanism that regulates lactate-mediated activity in immunosuppression has been described by Brand et al.: lactic acid production and subsequent acidosis inhibit nuclear factor of activated T cells (NFAT), the transcription factor that activates CD8+ T cells and in NK cells. As a result, there is a decrease in the production of IFNg, a cytokine that plays a crucial role in innate and adaptive immune activity [128,129].

### 5.3. Hypertension: Hippurate, Citrate, and Succinate

A nationwide population-based cohort study using a National Health Insurance research dataset determined a positive association between hypertension and subsequence BC development [130]. High blood pressure can decrease hippurate excretion, as established in the research about urinary metabolite phenotypes within-population samples from China, Japan, the United Kingdom, and the United States [131]. A further association between hippurate excretion and blood pressure has been established by spontaneously observing hypertensive rats’ urinary metabolome [132].

Citrate and succinate are intermediates of the tricarboxylic acid cycle (TCA), and their decreased levels in BC patients’ urine have been generally interpreted as a change in the relative weights of glycolysis vs. OxPhos in the tumoral cells [43,44,50,51,52,56,60,61,62]. However, their role in the systemic metabolism goes beyond their function in TCA. A succinate-specific G protein-coupled receptor was identified in blood vessels, cardiomyocytes, and kidney epithelia [133,134], suggesting that succinate can act as an extracellular signaling molecule. Urinary citrate chelates free Ca^2+^, thus protecting against Ca^2+^ oxalate crystallization and renal and bladder calculi formation. Kidney stone formation is associated with elevated risk for developing hypertension [135], and Khamaysi et al. suggested a molecular link between these two disease states because a protein complex mediates and regulates citrate and succinate transport, with the first associated with kidney stones formation and the second with hypertension [136]. In addition, urinary citrate concentration can be altered in patients subjected to radical cystectomy (RC) and subsequent urinary diversion, which is routinely practiced to treat high-risk NMIBC after the failure of intravesical therapy or to treat MIBC [137]. The incidence of renal stone formation in patients with this kind of surgery is relatively high, leading to a decreased urinary citrate level [137]. Taking all together, these results suggest that systemic pathways other than TCA heavily influence the urinary concentrations of these two metabolites.

### 5.4. Impaired Renal Function: Phenylacetylglutamine, Taurine, and Tyrosine

Up to 40% of patients with bladder cancer also have renal impairment [138]. Phenylacetylglutamine (PAGN) renal clearance decreased five-fold in severe renal impairment compared to subjects with normal renal function [139]. The link between PAGN concentration and renal dysfunction is based on the fact that, in humans, conjugation of phenylacetic acid (PAA) and glutamine to form PAGN increases from 45% in plasma to 90% in urine. These values indicate that 50% of urine PAGN derives from kidney conjugation of free plasma PAA and/or from the kidney’s preferential filtration of conjugated PAA [140]. Considering these data, decreases in urinary PAGN observed in different BC urinary metabolomic studies [46,49,60,63] can be partially linked to this comorbidity.

Taurine was the first metabolite proposed as a BC biomarker [44]. Other studies confirmed an increase in this compound in BC patients [78,89,99,100] and PCa patients’ urine [69], which precludes its definition as a specific BC biomarker. A study about the urinary metabolomic analysis of patients with acute heart failure identified taurine as the most significant variable to detect early renal injury [141]. More evidence is needed to separate the covariation in taurine excretion due to BC and renal impairment to determine the cause of its derangement in urine.

Tyrosine was observed to increase in the urine of BC patients in three studies and to decrease in one [47,56,60,63]. Both tyrosine and phenylalanine are excreted in small quantities in the urine of normal men and women, but in patients with advanced renal failure, the ratio of the clearance of these two amino acids to the glomerular filtration rate is increased [142]. This effect can be in part responsible for the change observed in the urinary tyrosine level.

As already pointed out by Van et al., these examples show the importance of eliminating the effects of kidney diseases and other urinary tract comorbidities when defining the urinary metabolic profile provoked by BC [143].

### 5.5. The Thrombosis Network: Citrate and Valine

At least 20% of cancer patients will suffer a thrombotic event during their lives, and thromboembolism is considered a leading cause of death for those receiving outpatient chemotherapy [144]. In fact, venous thromboembolism (VTE) may be the first manifestation of an occult tumor in an individual who would be otherwise considered healthy [145]. The reciprocal connection between cancer and thrombosis, known for more than one century, depends on the fact that, on the one hand, cancer cells support the formation of clots, while, on the other hand, coagulant proteins promote the growth and spread of cancer [145]. The principal mechanism by which malignancy induces a hypercoagulable state involves a transmembrane glycoprotein, tissue factor (TF), that activates the clotting cascade by interaction with the coagulation factor VII/VIIa on the cell surface [146].

The risk of VTE is particularly high in BC patients, with an incidence of 7.9 events per 100 patient-years in those with metastatic disease [147]. Patients who undergo RC and have TF-positive tumors have a three times higher risk of dying from their disease than TF-negative patients [148].

Several metabolomics studies have attempted to elucidate the molecular basis of thrombosis [149]. The metabolites most frequently shown to have altered levels are acetate, citrate, glucose, phenylalanine, valine, and 3-hydroxybutyrate. As in the cases analyzed above, at least two metabolites belonging to the BC consensus list of Table 2, citrate and valine, also appear as candidates for biomarkers of this systemic effect.

### 5.6. Cachexia and Sarcopenia: Acetylcarnitine, Carnitine, Citrate, Creatine, Glutamine, Lactate, Succinate, Taurine, Tyrosine, Uridine, and Valine

Cancer cells display an altered metabolism particularly well fitted to the attainment of sufficient energy and a high metabolic turnover rate. This high amount of energy arrives from the tumor biochemical pathways, but a “metabolic dictatorship” is also established over the whole organism to cope with the demand for substrates [150]. In this way, a network is established between different tissues, tumors, muscle and adipose, and organs, including the liver, pancreas, brain, and gut [151]. This network configures a global syndrome known as cachexia, which shows a high incidence among cancer patients and is directly related to inflammation: cytokines such as IL-6, TNF-α, IL-1β, and TGF-β induce inflammation and adipose tissue wasting [152,153].

Unlike starvation, in which liver and fat mass are lost but not lean body mass, cancer cachexia is characterized by loss of lean body mass (sarcopenia) with or without fat mass loss [154]. The prognostic role of sarcopenia in BC has been the subject of several studies in recent years. In general, it was observed that sarcopenia is associated with a poor prognosis in patients who underwent RC or advanced inoperable cases of BC [155,156]. Metabolically, sarcopenia is related to glucose, lipid, and protein metabolism [157]. Experimental evidence suggests that these metabolic alterations arise from changes induced in the host metabolism by the tumor and not from the metabolic activity of the tumor itself [101].

Yang et al. analyzed the serum and urine metabolic profiles of patients with cancer cachexia, pre-cachexia (weight loss < 5%), weight-stable cancer, and healthy controls and found that 45 metabolites were responsible for differences among all these groups [158]. Nine metabolites of those indicated in Table 2 as biomarkers of BC coincide with those listed in the study by Yang et al.: carnitine, citrate, creatine, glutamine, lactate, succinate, tyrosine, uridine, and valine. For example, lactate levels differed among the testing groups: they were increased in the weight-stable cancer group with respect to both healthy and cancer cachexia groups but lower than in the pre-cachexia group [158]. The fact that lactate concentration shows these variations makes it a very unspecific marker and highly dependent on the composition of the BC group in terms of body mass stability or weight loss.

In another study, Stretch et al. used metabolomics to predict the skeletal muscle and fat mass changes in patients with advanced cancer [159]. They have listed a series of controls to separate cancer-specific metabolic discriminants from those associated with lean and fat mass variations. Participants must be enrolled in the fasted state, stratified for protein intake, or provided a standardized protein intake before the measurements and divided into predefined lean and fat mass [159]. As in the case of the study by Yang et al., many metabolites from Table 2 were discovered to be related to the variation in body mass, including acetylcarnitine, taurine, creatine, succinate, lactate, and tyrosine.

## 6. Final Remarks

Despite the great expectation of using urine as a particularly favored biofluid for discovering BC markers, no metabolite or set of metabolites proposed to be altered in this biofluid have yet demonstrated sufficient sensitivity and specificity to replace cystoscopy for BC diagnosis [160,161,162]. Although the number of studies is still low compared with those dedicated to other diseases, this is not the only drawback. Some of the problems underlying this failure were discussed in this review. Care must be taken in standardizing the composition of the groups, their geographical origin and diets, the type of tumor considered, and adjusting the technical details finely. A series of methodological recommendations about implementing NMR and MS-based metabolomics can be found in the review by Dinges et al. [113].

Concerning the ultimate causes of the observed alterations in the urinary metabolic profile provoked by BC, we have emphasized the systemic response of the “cancerized” body. This analysis complements most metabolomics BC papers’ explanations, primarily based on tumor biochemical imbalance reflected directly in the urine. While the contribution of the metabolite exchange between the tumor and urine can play a significant role for low concentrated compounds, the magnitude of the observed changes in metabolic levels and the large number of tightly interconnected systemic responses to cancer evidenced in recent years increase the likelihood that it is the whole-body reaction that prevails. When the biological understanding of cancer metabolic pathways is pursued, the tumor metabolism and the systemic response must be considered simultaneously to rationalize the observed metabolic variations. This may not be sufficient, however, and it may be essential to combine results with other omics fields, such as genomics, transcriptomics, and proteomics, and different types of samples, such as tissue and serum.

The search for specific metabolites whose alteration represents what occurs inside tumor cells will require focusing attention on less concentrated and more specific compounds. In fact, the concept of using urine as a liquid biopsy refers mainly to the detection of cell-free tumor nucleic acids and other tumor-derived materials [163]. An alternative is to use urinary extracellular vesicles containing miRNAs and IncRNAs, promising diagnostic biomarkers [164]. In this scenario, the role of metabolomics is yet to be discovered.

Quandt et al. reviewed most of the “pros” and “cons” for the use of tumor biopsies vs. peripheral biomarkers for their implementation into clinical decision-making for cancer immunotherapy [165]. They fit perfectly for evaluating the potentials and the shortcomings of using urine to discover BC biomarkers. Tissue biopsies represent the gold standard because they allow the in situ analysis of the primary site of interest and provide the spatial resolution of the tumor microenvironment. On the contrary, the method is highly invasive; patients usually do not lend themselves to the necessary surgical procedure; there is a need for serial assessment, and the results are heavily dependent on the tumor heterogeneity. Peripheral biofluids, like serum or urine, can provide markers with a minimally invasive procedure; serial assessment is easy; they can be monitored over the entirety life; samples are prepared at a reduced cost, and the material is easily accessible from patients and healthy volunteers. The systemic signature they reflect can alleviate tumor heterogeneity because the global response can be a homogenizing feature. There are many disadvantages, including the lack of spatial resolution, the lack of knowledge of the extent to which biofluids reflect the host–tumor interaction or the global systemic consequences, and if they genuinely represent tumor heterogeneity. This last constraint may be a limitation when searching for the best treatment for a BC patient based on his metabolic profile.

**Scheme 2:**
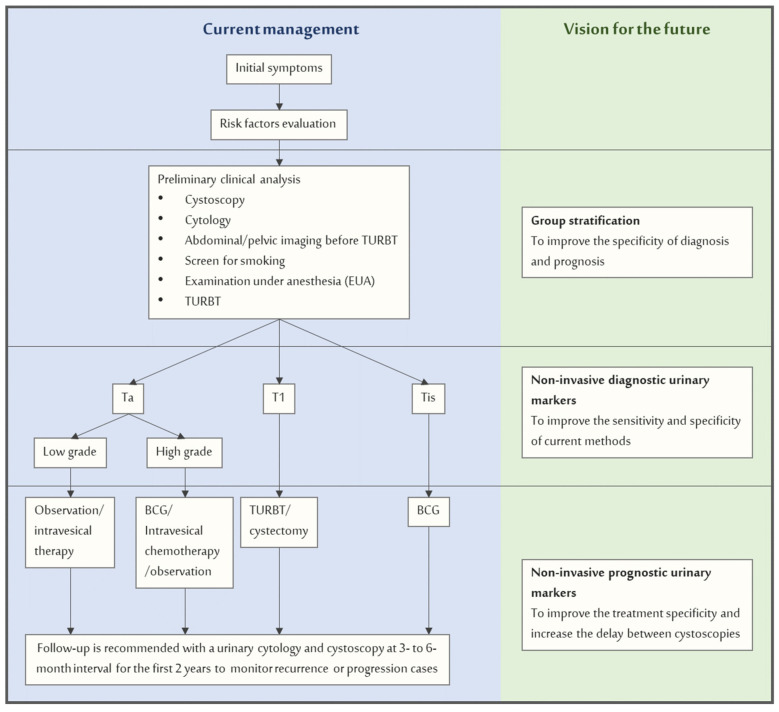
A new paradigm of NMIBC management exploiting non-invasive metabolic biomarkers as a complement to the current clinical practice guidelines [168]. Abbreviations: BCG: Bacillus Calmette–Guèrin, TURBT: Trans Urethral Resection of Bladder Tumor.

A new paradigm of disease management can be foreseen by introducing non-invasive biomarkers into the usual clinical practice of NMIBC treatment and follow-up. It includes improvements in early diagnosis, prognosis, choice of efficient treatment, and follow-up during the entire life span (Scheme 2). The main benefits will be the reduced cost of the treatment and follow-up and the positive influence on patients’ quality of life due to the non-invasiveness character of these markers. Their inclusion acquires more significance considering the actual emergency dictated by the COVID-19 pandemic, which causes a considerable increase in waiting times for cystoscopies, delaying diagnoses and lessening the surveillance quality [166]. For this reason, it was proposed that urinary biomarkers in diagnostic and prognosis must expand their role in disease management [167]. The discovery of urinary metabolic markers that can replace the current gold-standard test or improve its selectivity remains a significant challenge for scientists working in metabolomics. Among other hurdles, bridging the gap between basic research and clinical practice remains a huge obstacle to overcome, and this is the direction in which it is most important to redouble our efforts.

## Figures and Tables

**Table 1:** List of research works on urinary metabolomics for the discovery of BC diagnostic biomarkers.

References	Platform	Control Group (CTRL)			Bladder Cancer Patients (BC)		
		Type	%M ^a^	Age	Type	%M ^a^	Age
Issaq et al., 2008 [42]	LC-MS	Healthy (48)	44	59 (20–86)	MIBC + NMIBC (41)	88	76 (51–93)
Pasikanti et al., 2010 [43]	GC-TOF	non-BC (51)	55	67 ± 12	NMIBC (24)	83	61 ± 12
Srivastava et al., 2010 [44]	^1^H-NMR	Healthy (37), UTI ^b^ (31) bladder stone (2)	41	33 ± 15	NMIBC (33)	100	45 ± 25
Kim et al., 2010 [45]	GC-MS	Healthy (8)	100	NR ^c^	NMIBC (8)	100	47–78
Huang et al., 2011 [46]	LC-MS	Healthy (32)	56	53 (46–67)	NMIBC (27)	70	56 (42–71)
Putluri et al., 2011 [47]	LC-MS	Healthy (13)	62	53 ± 11	MIBC + NMIBC (13)	85	61 ± 14
	LC-MS	Benign patients (16)	75	69 ± 12	MIBC + NMIBC (28)	82	66 ± 13
	LC-MS	Benign patients (11)	NR	68 ± 14	MIBC + NMIBC (34)	NR	71 ± 10
	LC-MS	Healthy (11)	45	NR	MIBC + NMIBC (8)	50	NR
Gamagedara et al., 2012 [48]	LC-MS/MS	No-evidence-of-malignancy (NEM) (12)	NR	NR	BC ^d^ (11)	NR	NR
Huang et al., 2013 [49]	LC-MS	Healthy (24)	62	50 (26–65)	MIBC + NMIBC (19)	74	60 (45–74)
	LC-MS	Kidney cancer (25)	60	55 (27–71)	MIBC + NMIBC (19)	74	60 (45–74)
Pasikanti et al., 2013 [50]	GC-TOF	non-BC (61)	59	60 ± 13	NMIBC (38)	84	68 ± 11
Wittmann et al., 2014 [51]	LC and CG MS	non-BC (266)	64	64	MIBC + NMIBC (66)	85	67
Jin et al., 2014 [52]	LC-MS	Healthy (69), benign HU ^e^ (52)	64	64 ± 9	MIBC + NMIBC (138)	81	66 ± 13
Peng et al., 2014 [53]	LC-QTOFMS	Hernia (68), UTI ^b^ or HU (31)	91	62 ± 12	MIBC + NMIBC (91)	70	68 ± 13
Shen et al., 2015 [54]	LC-MS	Healthy (21)	57	54 ± 19	MIBC + NMIBC (23)	78	65 ± 13
Shao et al., 2017 [55]	UPLC-TOF	Hernia (65)	95	65 ± 13	MIBC + NMIBC (87)	62	68 ± 14
Zhou et al., 2017 [56]	GC-MS	Healthy (35)	66	63 ± 8	MIBC + NMIBC (50)	70	63 ± 12
Mpanga et al., 2018 [57]	LC-MS	Healthy (40)	55	60 (53–81)	BC (40)	50	62 (50–87)
Cheng et al., 2018 [58]	LC-HRMS	Healthy (78)	78	59 ± 11	NMIBC (54)	78	62 ± 13
Liu et al., 2018 [59]	LC-HRMS	Healthy (203)	48	20–60	NMIBC (110)	64	64 ± 13
Loras et al., 2018 [60]	UPLC-TOF-MS	NMIBC after TURBT (18)	53	67 ± 11	NMIBC before TURBT (18)	53	67 ± 11
Loras et al., 2019 [61]	^1^H-NMR	MIBC + NMIBC after TURBT (21)	67	69 ± 10	MIBC + NMIBC before TURBT (12)		
Loras et al., 2019 [62]	^1^H-NMR	MIBC + NMIBC after TURBT (17)	59	71 ± 9	MIBC + NMIBC before TURBT (13)		
Jacyna et al., 2019 [63]	^1^H-NMR, GC-MS, HPLC-MS	Healthy (24)	75	64 ± 10	MIBC (24)	75	65 ± 12
Wang et al., 2019 [64]	UPLC-MS	Healthy (98)	59	55 (20–91)	NMIBC (53)	77	62 (33–87)
		RCC ^f^ (64)	75	53 (14–82)	NMIBC (146)	77	62 (33–87)
Łuczykowski et al., 2021 [65]	SPME-LC-MS	Healthy (24)	75	64 ± 10	MIBC (24)	76	65 ± 13
Pinto et al., 2021 [66]	HS-SPME-GC-MS	Cancer-free (56)	71	52 (45–66)	MIBC + NMIBC (53)	74	69 (43–87)
Lin et al., 2021 [67]	GC-MS	Hernia (61)	95	65 ± 12	NMIBC (63)	71	67 ± 13

^a^ Male percentage. ^b^ Urinary-Tract-Infection. ^c^ Not reported. ^d^ Tumor type not specified. ^e^ Hematuria. ^f^ Renal cell carcinoma.

**Table 2:** Urinary metabolites proposed for bladder cancer diagnosis that were found to be perturbed in at least three studies.

Metabolite	BC/CTRL	References
hippuric acid	↓↓↓↓↓↓↓↓↓↓?? ^a^	[44,46,47,48,49,56,60,61,62,63,65]
citric acid	↓↓↓↓??	[43,44,50,60,61,62]
gluconic acid	↑↑↓↓↓	[43,50,51,57,65]
lactic acid	↑↑↑↑?	[50,51,56,61,63]
taurine	↑↑↑??	[44,48,49,61,62]
uridine	↑↑↑↓?	[43,45,50,53,63]
valine	↑↑↑??	[43,47,51,61,62]
phenylacetylglutamine	↑↓↓↓	[46,49,60,63]
succinate	↑↓↓?	[51,52,56,61]
tyrosine	↑↑↑↓	[47,56,60,63]
carnitine	↑↑↓↓	[46,47,52,60]
ribitol	↑↓↓↓	[43,50,56,67]
creatine	↑↓↓?	[47,51,60,62]
p-cresol	↑↑↓	[50,56,66]
acetyl-carnitine	↑↑↓	[46,51,60]
5-hydroxyindoleacetic acid	↑↓↓	[53,60,63]
fructose	↑↓↓	[43,51,67]
glutamine	↑??	[61,62,63]
histidine	↑??	[47,62,63]
erythritol	↑↑↑	[50,63,67]

^a^ Each symbol refers to a single study; “↑” “↓” symbols represent higher and lower alterations in metabolite concentrations in cancer versus healthy control group, respectively; “?”: direction of variation not reported.

**Table 3:** Putative BC urinary biomarkers and their predicted changes in total mass between BC and control groups.

Metabolite	Normal Range ^a^	Fold Change	ΔMass ^b^ (mg)
gluconic acid	21.5 (8.1–38.8)	11.0	177.1
p-cresol	46.0 (1.2–118.9)	1.7	14.6
lactic acid	12.3 ± 6.2	3.1	10.0
erythritol	33.4 (6.8–64.0)	1.2	3.4
acetyl-carnitine	2.8 (0.6–7.5)	2.4	3.3
taurine	4.00–159.98	2.5	3.2
uridine	0.994 (0.637–1.351)	1.8	0.8
valine	3.0 ± 1.0	1.5	0.8
5-hydroxyindoleacetic acid	2.9 (0.4–5.8)	1.3	0.7
ribitol	3.5 ± 2.24	0.7	−0.6
succinate	5.6 ± 3.8	0.6	−1.0
creatine	46 (3–448)	0.3	−17.7
phenylacetylglutamine	47.03 (3.84–85.51)	0.5	−26.1
fructose	150 (100–200) ^c^	0.7	−37.5
citric acid	242.0 ± 129.6	0.8	−39.1
hippuric acid	257 (20–770)	0.5	−96.7

^a^ Values in μM/mM of Creatinine. ^b^ Average mass difference between BC and normal subjects urine and calculated using the following formula: $\Delta \mathrm{mass}=\mathrm{NR}*10^{-3}\times C_{c}\times V_{u}\times \mathrm{MW}\times \left(\mathrm{FC}-1\right)$, where NR is the average value reported in this table; C_c_ is the average Creatinine concentration (14 mM); V_u_ is the average urine volume (0.3 L); MW is the metabolite molecular weight, and FC is the fold change. ^c^ M/24 h.
